# Prevalence of Anemia and Its Associated Factors among Pregnant Women Attending Antenatal Care in Health Institutions of Arba Minch Town, Gamo Gofa Zone, Ethiopia: A Cross-Sectional Study

**DOI:** 10.1155/2016/1073192

**Published:** 2016-02-22

**Authors:** Alemayehu Bekele, Marelign Tilahun, Aleme Mekuria

**Affiliations:** ^1^Department of Public Health Nursing, Arba Minch College of Health Sciences, P.O. Box 155, Arba Minch, Ethiopia; ^2^Department of Public Health, College of Health Sciences, Debre Tabor University, P.O. Box 272, Debre Tabor, Ethiopia

## Abstract

*Background*. Anemia during pregnancy is a major cause of morbidity and mortality of pregnant women in developing countries and has both maternal and fetal consequences. Despite its known serious effect on health, there is very little research based evidence on this vital public health problem in Gamo Gofa zone in general and in Arba Minch town of Southern Ethiopia in particular. Therefore, this study aims to assess the prevalence and factors associated with anemia among pregnant women attending antenatal care in health institutions of Arba Minch town, Gamo Gofa zone, Southern Ethiopia.* Method*. Institution-based, cross-sectional study was conducted from February 16 to April 8, 2015, among 332 pregnant women who attended antenatal care at government health institutions of Arba Minch town. Interviewer-administered questionnaire supplemented by laboratory tests was used to obtain the data. Bivariate and multivariate logistic regressions were used to identify predictors of anemia.* Result*. The prevalence of anemia among antenatal care attendant pregnant women of Arba Minch town was 32.8%. Low average monthly income of the family (AOR = 4.0; 95% CI: 5.62–11.01), having birth interval less than two years (AOR = 3.1; 95% CI: 6.01, 10.23), iron supplementation (AOR = 2.31; 95% CI: 7.21, 9.31), and family size >2 (AOR = 2.8; 95% CI: 1.17, 6.81) were found to be independent predictors of anemia in pregnancy.* Conclusion*. Anemia is found to be a moderate public health problem in the study area. Low average monthly income, birth interval less than two years, iron supplementation, and large family size were found to be risk factors for anemia in pregnancy. Awareness creation towards birth spacing, nutritional counselling on consumption of iron-rich foods, and iron supplementation are recommended to prevent anemia among pregnant women with special emphasis on those having low income and large family size.

## 1. Background 

Anemia is defined as a decrease in the concentration of circulating red blood cells or in the haemoglobin concentration and a concomitant impaired capacity to transport oxygen. It has multiple precipitating factors that can occur in isolation but more frequently cooccur. These factors may be genetic, such as haemoglobinopathies; infectious diseases, such as malaria, intestinal helminths, and chronic infection or nutritional deficiency, which includes iron deficiency as well as deficiencies of other vitamins and minerals, such as folate, vitamins A and B12, and copper [1].

Anemia is a global public health problem affecting both developing and developed countries with major consequences on human health as well as social and economic development. It occurs at all stages of the life cycle but is more prevalent in pregnant women and young children [2]. Although the prevalence of anemia is estimated at 9% in countries with high development, in countries with low development the prevalence is 43%. Children and women of reproductive age are most at risk, with global anemia prevalence estimates of 47% in children younger than 5 years, 42% in pregnant women, and 30% in nonpregnant women aged 15–49 years and with Africa and Asia accounting for more than 85% of the absolute anemia burden in high risk groups [3].

Anemia during pregnancy is a major cause of morbidity and mortality of pregnant women in developing countries and has both maternal and fetal consequences. Anemia during pregnancy is considered severe when haemoglobin concentration is less than 7.0 g/dL, moderate when haemoglobin falls between 7.0 and 9.9 g/dL, and mild when haemoglobin concentration is from 10.0 to 11 g/dL [1, 3–5].

Low maternal haemoglobin levels are associated with increased risk of preterm delivery, Low Birth Weight (LBW) babies, APGAR score <5 at 1 min, and intrauterine growth retardation (IUGR). Haemoglobin adjusted for altitude and smoking status in Ethiopia shows that 22% of pregnant women in Ethiopia are anemic and the prevalence varies by residence and educational and wealth status of women. The Health Sector Development Plan IV (HSDP IV) in Ethiopia targets reducing the national level of anemia to 12% [3, 6].

Nutritional, genetic, and infectious diseases are contributing factors for anemia. However, iron deficiency is the cause of 75% of anemia cases. The understanding of how these factors vary by geography, level of development, and other social and economic factors will make it easier to design interventions that are more effective and integrative in addressing multiple contributing factors at the same time [1, 7].

In Ethiopia, even though the HSDP IV target is to reduce anemia prevalence nationally to 12 percent, still anemia is severe problem and affecting 22% of pregnant mothers [6, 8]. Given the multifactorial nature of this disease, correcting anemia often requires an integrated approach. In order to effectively combat anemia, the contributing factors must be identified and addressed. The availability of local prevalence statistics has a major role in the management and control of anemia in pregnancy. Besides the limited studies done in Ethiopia, the prevalence of anemia in pregnant women in our study area is not well known so far. Therefore, this study aims at assessing the prevalence of anemia and its associated factors among pregnant women attending ANC in health institutions of Arba Minch town, Gamo Gofa zone, Southern Ethiopia.

## 2. Methods 

### 2.1. Study Area

The study was conducted at Arba Minch General Hospital, Arba Minch, and Secha Health Centers in Arba Minch town, Ethiopia. All the three health institutions are located in Arba Minch town which is the capital of Gamo Gofa zone. The catchment area of the two health centres is Arba Minch town and that of the hospital is Gamo Gofa zone. The altitude of the area is 1285 m (4216 ft) above sea level. The total population of the town is about 103,965 people; by using conversion factor for 2014/15 of SNNPR pregnant women in the town it is expected to be 3296 [9]. The three health institutions are the only institutions which provide ANC service for pregnant women in Arba Minch town. The ANC service is provided by midwives who had got special training on focused antenatal care model.

### 2.2. Study Design

Institution-based, cross-sectional study design was employed.

### 2.3. Study Participants

The study participants for this study were pregnant women who were attending antenatal care at the selected health institutions during the study period.

### 2.4. Inclusion Criteria

Pregnant women who reside in Arba Minch for more than six months and who came for ANC during the study period were included in the study.

### 2.5. Exclusion Criteria

Pregnant women who were seriously ill during the survey were excluded.

### 2.6. Sample Size and Sampling Procedure

A sample size of 332 was calculated using single proportion formula assuming 16.6% proportion of anemia in pregnancy (from previous study in Ethiopia [10]) at a 95% confidence limit, 80% power, and 4% margin of error and adding 10 % as contingency for nonresponse.

**Figure 1:**
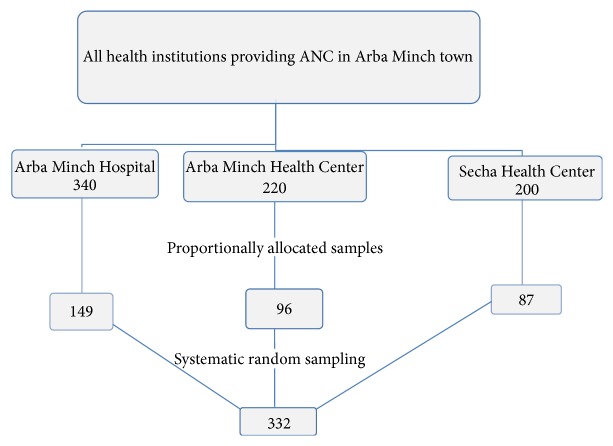
Schematic presentation of sampling procedure in public health institutions of Arba Minch town, 2014 (*n* = 332).

We have reviewed the last year records of monthly flow of pregnant women for ANC utilization in the three health institutions (340 attendees from Arba Minch General Hospital, 220 from Arba Minch Health Center, and 200 attendees from Secha Health Center). The calculated sample size (332) was proportionally allocated to these three health institutions based on the above records: 149 pregnant women for Arba Minch General Hospital, 96 for Arba Minch Health Centre, and 87 for Secha Health Center. Then, after systematic random sampling technique was employed, for instance, in the case of Arba Minch Hospital, the total monthly number of mothers who attended ANC (340) was divided into the current allocated sample size (149) to get the interval that is two, and then lottery method was used to get the random start. Therefore, randomly selected starting point was number two. The interview was started from the second mother, and then every two mothers were interviewed till the allocated sample was achieved. The same method was applied across the rest centres (Figure 1).

### 2.7. Data Collection

A structured pretested interviewer-administered questionnaire was used to obtain sociodemographic information and present and past obstetric history in pregnant women. To obtain dietary habit, standard food frequency questionnaire adjusted for local food item was adapted and used to assess the usual intake of various food groups for the past one month with their respective consumption frequency [10]. The questionnaire was developed in English and then translated into Amharic language for simplicity then back-translated to English language for its consistency by two different language expert individuals who speak both English and Amharic fluently. Pretesting of the questionnaire was done on 5% of the sample size among ANC attendees who were not included in the study; that was a week before commencement of the actual data collection. To ensure reliable data collection and attain standardization and maximize interviewer reliability, midwives who speak both Amharic and the local language (Gamogna) were recruited and given training on data collection procedure. Exit interview was done. The data collectors were regularly supervised for proper data collection; all the questionnaires were checked for completeness and consistency in daily basis.

### 2.8. Specimen Collection and Processing

The specimen collection process in the three health institutions was carried out by two trained laboratory technologists. Each step of specimen collection, processing, and analysis was supervised by experienced and trained laboratory technologist supervisors. The blood for hematocrit/packed cell volume (PCV) measurement was done based on the Standard Operational Procedures (SOPs).

A venous blood sample was taken from the study participants; using heparinized hematocrit tube, three-fourths of the tube was filled and labeled with identification number. The capillary tube after being sealed at one end was centrifuged in the microhematocrit centrifuge at 10,000 g for 5 minutes. Then, the result was read using hematocrit reader.

Stool samples were collected by using a clean and labeled container from the study participants. A portion of the stool was processed with direct microscopic technique to detect intestinal parasites immediately. For detection of helminths, eggs, larvae, and cysts of protozoan parasites, the samples were examined microscopically first with 10x and then with 40x objective. The remaining part of the sample was emulsified in a 10% formalin solution.

Stool examinations were done using formal ether concentration technique, which is considered the most sensitive for most intestinal helminthes. The same method was carried out across all centres.

The hematocrit values in our study area were adjusted in line with the WHO graded adjustment for altitudes; since the altitude of our study area is 4216 feet above sea level, the normal increase for hematocrit values related to long-term exposure is 1%. Therefore, the value is adjusted with the given range [11].

### 2.9. Operational Definitions and Definition of Terms

*Anemia in Pregnancy*. It is when the hematocrit value for a pregnant woman is less than 33% irrespective of her gestational age [11].

*Public Health Importance of Anemia*. It is a mild public health problem, when prevalence of anemia is <20%; a moderate public health problem, when the prevalence of anemia is between 20 and 40%; a severe public health problem, when the prevalence of anemia is >40% [11].

*Mild Anemia*. Hematocrit value is ≥30% and <33%.

*Moderate Anemia*. Hematocrit value is ≥21% and <30%.

*Severe Anemia*. Hematocrit value is from <21%.

*Eating Habits*. Different eating habits include eating animal foods, green leafy vegetables, taking fruit after meal, and drinking tea/coffee; this is measured by using food frequency questioner [10].

*Permanent Resident*. Pregnant women lived at least six months in the study area.

*Monthly Income of the Family*. Monthly income of the family is <1000 Ethiopian Birr (low), 1000–2575 Ethiopian Birr (medium), and >2575 Ethiopian Birr (high).

### 2.10. Data Analysis

Data was entered to Epi Info version 3.5.3 software and then exported to SPSS version 20 statistical packages for analysis. Descriptive statistics were done and summarized by frequencies and proportion for categorical predictors. The presence of association was assessed using bivariate analysis and associations with a *p* value <0.05 considered as statistically significant. Multivariate logistic regression was used to control confounding effects and the strength of association was estimated in odds ratio and its 95% confidence interval.

### 2.11. Ethical Consideration

Ethical approval was obtained from Joint MPH program, Arba Minch University, and Addis Continental Institute of Public Health Institutional Ethical Review Committee. Official letter to the hospital and health centres was obtained from College of Medicine and Health Sciences, Arba Minch University. To ensure confidentiality, it was anonymous type whereby names of the study subjects were not written on the questionnaire. Written consent from the study participants was obtained after they were briefed about the research intent and asked for their willingness to participate in the study. Their right of denial to participate in the study was also assured.

## 3. Result

### 3.1. Sociodemographic Characteristics of Study Subjects

A total of 332 representative ANC attendees participated in the study yielding the response rate of 100%.

The median age of the participants was 25 ± 4.28. Majority, 256 (77.1%), of the participants were within the age range of 20–29. Three hundred and twenty-five (97.9%) of the attendees were married but only four (1.2%) were single. With regard to religion, 174 (52.4%) were Protestants followed by Orthodox Christians, 147 (44.3%). Pertaining to educational status, 103 (31%) of the participants achieved secondary school and above. However, 62 (18.7%) had no formal education. The mean family size in this study group is 3.51 ± 1.66. One hundred and eighteen (35.5%) and 80 (24.1%) of the attendees had a family size of less than or equal to two and greater than or equal to five, respectively (Table 1).

### 3.2. Obstetrics and Medical History

Two hundred and seven (62.3%) of the ANC attendees had previous history of pregnancy. From those who had previous history of pregnancy, 51 (24.6%) and 63 (32.8%) had history of miscarriage or wilful abortion and at least history of one child, respectively. One hundred and twenty-five (69.1%) had their last births in health facilities. Fifteen (4.5%) of the ANC attendees had history of bleeding on the current pregnancy. One hundred and seventy-six (89.3%) had history of ANC follow-up in the previous pregnancy. From those who had history of birth, 28 (13.5%) and 179 (86.5%) had birth interval of less than two years and more than two years between the last and the current pregnancy, respectively.

More than half of the ANC attendees, 202 (60.8%), had history contraceptive use. More than quarter, 103 (31%), of the participants had history of malaria attack in the last one year. About 123 (38.0%) were using iron during the current pregnancy. Concerning gestational age, 48 (14.5%), 178 (53.6%), and 106 (31.9%) were in first, second, and third trimester of pregnancy, respectively (Table 2).

*Prevalence of Anemia*. About one-third, 109 (32.8%), of the 332 ANC attendees were anemic (hematocrit < 33%). From those who were anemic, the majority, 57 (52.3%), were mild (hematocrit value ≥ 30% and <33%) and 4 (3.7%) were severely anemic (hematocrit value < 21%). Eleven (3%) of the pregnant women were found to be HIV positive.* Giardia lamblia*, 30 (9%);* Entamoeba histolytica*, 5 (1.5%); hookworm, 2 (0.6%), were among intestinal parasites detected in the pregnant women (Table 3).

*Factors Associated with Anemia among Pregnant Women*. Factors associated with anemia in pregnancy were assessed. During the bivariate analysis, educational status, family size, iron supplementation on current pregnancy, and monthly income had statistically significant association with anemia in pregnancy (Tables 4 [Table tab5]–6).

In multivariate logistic regression, monthly income of the family (AOR = 4.0; 95% CI: 5.62–11.01), family size (AOR = 2.8; 95% CI: 1.17–6.8), birth interval (AOR = 3.1; 95% CI: 6.01, 10.23), iron tablet supplementation (AOR = 2.31; 95% CI: 7.21, 9.31), and eating food made from “*Enset*” and its products (AOR = 5.11; 95% CI: 16.18, 21.35) were found to be independent predictors of anemia in pregnancy (Table 7).

## 4. Discussion 

The current study assessed the prevalence of anemia and its associated risk factors among pregnant women attending ANC in government institutions of Arba Minch town, Gamo Gofa zone, Southern Ethiopia. The overall prevalence of anemia among pregnant women attending ANC in the current study was found to be 32.8% which is lower than a study conducted in India (87–100%), Boditi (61.6%), and Gode town, Eastern Ethiopia (56.8%) [12–15]. This discrepancy could be resulting from geographical variation of factors across different areas and due to time gap between the current study and the 2011 Ethiopian Demographic and Health Survey.

However, the prevalence of anemia in the current study was found to be higher as compared to the study conducted in Addis Ababa (21.3%) and Gondar Northwest Ethiopia (16.6%) [10, 14, 16]. In addition, the current prevalence of anemia is also higher than the national anemia prevalence (22%) [6]. This might be attributed to the fact that the majority of the participants in the current study consume plant based foods as a staple food which is rich in nonheme iron with bioavailability of not more than 10%. The high consumption of tea and coffee in the study area might reduce the bioavailability of the nonheme iron from plant based staple foods.

In the current study among the pregnant women, mild anemia was found to be common and followed by moderate anemia. Consistent result was reported from studies conducted in some African countries and elsewhere in the world [12–15, 17–21].

Monthly income was significantly associated with anemia in pregnancy. Pregnant women who had low monthly family income (less than 2575 Ethiopian Birr) were four times more likely to be anemic as compared to those with high monthly family income (greater than 2575 Ethiopian Birr). This is in agreement with some studies [10, 14]. This could be explained by the reality that more than 57% of the total expenditure among Ethiopians is spent on food [15, 22]. Hence, pregnant women with low income groups could not get adequate nutrition so that they were at risk of anemia.

Pregnant women having birth interval less than two years were at higher risk of becoming anemic as compared to those with birth interval more than two years. This finding is consistent with a study conducted in Saudi Arabia [23]. This might be related with decreased iron store of women due to occurrence of pregnancy in quick succession between subsequent pregnancies.

Pregnant women who have had no iron supplementation on the current pregnancy were in about two times higher risk of developing anemia as compared to those who have had iron supplementation. This finding is consistent with the findings from Gode town (Eastern Ethiopia) and Vietnam [15, 24], which indicated that lack of iron supplementation was among the most significant risk factors for developing anemia during pregnancy. This is likely due to the fact that the requirement for iron increases for pregnant women as compared to nonpregnant women; this is associated with the reality that blood volume increases by 50% during pregnancy and the requirement of iron to growing fetus and placenta. Therefore, supplementation of iron during pregnancy is crucial to fulfil this need.

In this study, family size was also significantly associated with anemia; pregnant women with family size greater than 5 were at higher risk of developing anemia than those with family size less than five. This finding is comparable with a study conducted in Shala woreda (West Arsi) in which the prevalence of anemia was higher among women with family size >5 as compared to their counterparts [25]. The direct relationship of family size with anemia in this study could be associated with food insecurity for large family size.

*Study Limitations*. This study is limited by its cross-sectional nature, whereby it may not explain the temporal relationship between the outcome variable and some explanatory variables; this limits interpretation of the estimated associations. Recall bias might be introduced on food frequency. Thus, the findings of this study should be interpreted within these limitations.

## 5. Conclusion 

The overall prevalence of anemia among women attending ANC in government health institutions of Arba Minch town was 32.8%. Anemia is a moderate public health problem in Arba Minch, which is by far higher than the national prevalence, 22%. Monthly income, family size, birth interval, and iron supplementation were significantly associated with anemia. We recommend awareness creation on birth spacing and nutritional counselling on consumption of iron-rich foods and iron supplementation to prevent anemia among pregnant women with special emphasis on those from low income group and large family size.

## Figures and Tables

**Table 1:** Sociodemographic characteristics of ANC attendees in government health institutions of Arba Minch town, February to April 2015 (*n* = 332).

Variables	Number	%
Age		
15–19	32	9.6
20–29	256	77.1
30 and above	44	13.3
Marital status		
Single	4	1.2
Married	325	97.9
Divorced	3	0.9
Religion		
Orthodox	147	44.3
Protestant	174	52.4
Muslim	11	3.3
Educational status		
No formal education	62	18.7
Primary	88	26.5
Secondary	79	23.8
Above secondary	103	31
Family size		
≤2	118	35.5
3-4	134	40.4
≥5	80	24.1
Ethnicity		
Gamo	215	64.8
Wolayita	31	9.3
Amhara	35	10.5
Gofa	18	5.4
Others^*∗*^	33	10

^*∗*^Gurage, Oromo, Tigray, Konso, Burji, Derashe, Ari, Gidicho, Zayise, Oyida, Amaro, and Sidama.

**Table 2:** Obstetrics related characteristics among ANC attendees in health institutions of Arba Minch town, February to April 2015 (*n* = 332).

Variables	Number	%
History of previous pregnancy		
Yes	207	62.3
No	125	37.7
History of abortion		
Yes	51	24.6
No	156	75.4
Number of children		
1	63	32.8
2-3	102	53.1
≥4	27	14.1
Birth interval between the last and current		
Primigravida	125	37.7
<2 years	28	13.5
>2 years	179	86.5
Parity		
Nullipara (0)	125	37.6
Primipara (1)	28	8.4
Multipara (2–4)	159	47.9
Grand multipara (≥5)	30	9.0
Gestational age		
1st trimester	48	14.5
2nd trimester	178	53.6
3rd trimester	103	31.9
Place of delivery of previous pregnancy		
Home	64	30.9
Health facility	143	69.1
ANC follow-up in previous pregnancy		
Yes	176	89.3
No	21	10.7
Bleeding on current pregnancy		
Yes	15	4.5
No	317	95.5
Contraceptive use		
Yes	202	60.8
No	130	39.2
Malaria in the last one year		
Yes	103	31
No	229	69
Iron supplementation on current pregnancy		
Yes	123	37
No	209	63

**Table 3:** Laboratory findings of ANC attendees in government health institutions of Arba Minch town, February to April 2015 (*n* = 332).

Variable	Number	%
HIV serostatus		
Negative	321	96.7
Positive	11	3.3
Stool examination		
*Giardia lamblia*	30	9
Hookworm	2	0.6
*Ascaris lumbricoides*	1	0.3
*Entamoeba histolytica*	5	1.5
*Taenia* species	2	0.6
No parasite	291	87.7

**Table 4:** Sociodemographic factors associated with anemia in pregnancy among ANC attendees in government health institutions of Arba Minch town from February to April 2015 (*n* = 332).

Variables	Anemia		COR (95% CI)
	Yes	No	
Occupation			
House wife	56 (33.5%)	111 (66.5%)	1.00
Civil servant	13 (20.3)	51 (79.7%)	0.5 (0.25–1.01)
Merchant	18 (37.5%)	30 (62.5%)	1.2 (0.61–2.32)
Day labourer	12 (60%)	8 (40%)	**2.9 (1.15–7.69)**
Others	10 (30.3%)	23 (69.7%)	0.9 (0.38–1.94)
Monthly income			
<1000 ETB	61 (41.2%)	87 (58.8)	**3 (7.41–10.67)**
1000–2575 ETB	38 (36.2%)	67 (63.8%)	0.81 (0.48–1.35)
>2575 ETB	10 (12.7%)	69 (87.3%)	** 1**
Educational status			
Illiterate	29 (46.8%)	33 (53.2%)	1.00
Primary	29 (33%)	59 (67%)	0.44 (0.18–1.05)
Secondary	26 (32.9%)	53 (67.1%)	0.66 (0.31–1.39)
Above secondary	25 (24.3%)	78 (75.7%)	**0.36 (0.18–0.71)**
Marital status			
Married	105 (32.3%)	220 (67.7%)	1.00
Others	4 (57.1%)	3 (42.9%)	2.8 (0.61–12.71)
Family size			
≤2	35 (29.7%)	83 (70.3%)	1.00
3-4	40 (29.9%)	94 (70.1%)	1.5 (0.69–3.18)
≥5	34 (42.5%)	46 (57.5%)	**2.1 (6.42–10.83) **

**Table 5 tab5:** Obstetrics factors associated with anemia in pregnancy among ANC attendees in government health institutions of Arba Minch town from February to April 2015 (*n* = 332).

Variables	Anemia		COR (95% CI)
	Yes	No	
Trimester			
First	12 (25%)	36 (75%)	1.00
Second	65 (36.5%)	113 (63.5%)	1.7 (0.84–3.55)
Third	32 (30.2%)	74 (69.8%)	1.3 (0.59–2.81)
History of malaria attack (last 1 year)			
No	78 (34.1%)	151 (65.9%)	1.00
Yes	31 (30.1%)	72 (69.9%)	0.83 (0.51–1.37)
Intestinal parasite on current pregnancy			
No	97 (33.3%)	194 (66.7%)	1.00
Yes	12 (29.3%)	29 (70.7%)	0.83 (0.41–1.69)
HIV serostatus			
Negative	106 (33%)	215 (67%)	1.00
Positive	3 (27.3%)	8 (72.7%)	0.76 (0.19–2.93)
Iron supplementation on current pregnancy			
No	73 (34.9%)	136 (65.1%)	**1.9 (6.4–9.10)**
Yes	36 (29.3%)	87 (70.7%)	1
Birth interval			
≤2 years	15 (53.6%)	13 (46.4%)	**2.3 (4.41–7.23)**
>2 years	52 (29.1%)	127 (70.9%)	1

**Table 6:** Dietary habits associated with anemia in pregnancy among ANC attendees in government health institutions of Arba Minch town from February to April 2015 (*n* = 332).

Variables	Anemia		COR (95% CI)
	Yes	No	
Eating food made from “*Enset*” and its products			
Twice/month	10 (43.5%)	13 (56.5%)	1.00
1-2 per week	25 (35.7%)	45 (64.3%)	0.72 (0.27–1.88)
3-4 per week	15 (17.9%)	69 (82.1%)	**0.28 (0.10–0.76)**
Once/day	38 (36.5%)	66 (63.5%)	0.75 (0.30–1.87)
>1 per day	21 (41.2%)	30 (58.8%)	**0.91 (0.34–2.46)**
Eating food made from cereals, grains			
2 times/wk	7 (43.8%)	9 (56.2%)	1.00
3-4/wk	7 (33.3%)	14 (66.7%)	0.64 (0.17–2.45)
Once/day	22 (18.3%)	98 (81.7%)	0.28 (0.09–0.86)
>1/day	73 (32.8%)	102 (67.2%)	0.92 (0.33–2.58)
Drinking tea or coffee			
2/month or less	6 (2.4%)	19 (76%)	1.00
1–4/wk	6 (20%)	24 (80%)	0.79 (0.22–2.85)
1/day	33 (25%)	99 (75%)	1.1 (0.38–2.86)
>1/day	64 (44.1%)	81 (55.9%)	2.5 (0.94–6.63)
Eating fruit			
≤2 wk	20 (45.5%)	24 (54.5%)	1.00
3-4/wk	25 (30.5%)	57 (69.5%)	0.53 (0.25–1.12)
1/day	38 (28.1%)	97 (71.9%)	0.47 (0.23–0.95)
>1/day	25 (35.7%)	45 (64.3%)	0.67 (0.31–1.44)
Eating beef, goat, chicken, or other kinds of organ meat			
Never	15 (23.1%)	50 (76.9%)	1.00
1-2/month	55 (37.4%)	92 (62.6%)	1.9 (1.02–3.88)
1-2/week	30 (34.9%)	56 (65.1%)	1.8 (0.86–3.69)
≥3/wk	9 (26.5%)	25 (73.5%)	1.2 (0.46–3.12)

**Table 7:** Multivariate logistic regression analysis results showing factors associated with anemia in pregnancy among ANC attendees in government health institutions of Arba Minch town from February to April 2015 (*n* = 332).

Variables	Anemia		COR (95% CI)	AOR (95% CI)
	Yes	No		
Monthly income				
<1000 Birr	61 (41.2%)	87 (58.8)	**3 (7.41–10.67)**	**4.0 (5.62–11.01)**
1000–2575 Birr	38 (36.2%)	67 (63.8%)	0.81 (0.48–1.35)	0.9 (0.54–1.75)
>2575 Birr	10 (12.7%)	69 (87.3%)	**1**	** 1**
Family size				
≤2	35 (29.7%)	83 (70.3%)	1.00	1.00
3-4	40 (29.9%)	94 (70.1%)	1.01 (0.59–1.73)	1.5 (0.69–3.18)
≥5	34 (42.5%)	46 (57.5%)	**2.1 (6.42–10.83)**	**2.8 (1.17–6.80)**
Iron supplementation on current pregnancy				
No	73 (34.9%)	136 (65.1%)	1.9 (6.4–9.10)	**2.31 (7.21, 9.31)**
Yes	36 (29.3%)	87 (70.7%)	1	** 1**
Birth interval				
≤2 years	15 (53.6%)	13 (46.4%)	**2.3 (4.41–7.23)**	**3.1 (6.01, 10.23)**
>2 years	52 (29.1%)	127 (70.9%)	0.81 (0.49–1.32)	** 1**
Eating food made from “*Enset*” and its products				
Twice/month	10 (43.5%)	13 (56.5%)	1.00	**1.00**
1-2 per week	25 (35.7%)	45 (64.3%)	0.72 (0.27–1.88)	**0.22 (0.07–0.73)**
3-4 per week	15 (17.9%)	69 (82.1%)	**0.28 (0.10–0.76)**	**0.12 (0.03–0.39)**
Once/day	38 (36.5%)	66 (63.5%)	0.75 (0.30–1.87)	0.36 (0.12–1.11)
>1 per day	21 (41.2%)	30 (58.8%)	**0.91 (0.34–2.46)**	**0.17 (0.05–0.62)**
